# Correction: Solution processed high performance perovskite solar cells based on a silver nanowire-titanium dioxide hybrid top electrode

**DOI:** 10.1039/d2ra90129c

**Published:** 2022-12-16

**Authors:** Khalid Mahmood, Hafiz Husnain Akhtar, Haji Ghulam Qutab, Naveed Ramzan, Rabia Sharif, Abdul Rehman, Arshi Khalid, Muhammad Taqi Mehran

**Affiliations:** Department of Chemical & Polymer Engineering, University of Engineering & Technology Lahore, Faisalabad Campus 3½ Km. Khurrianwala – Makkuana By-Pass Faisalabad Pakistan khalid@uet.edu.pk; Department of Chemical Engineering (ChE), University of Engineering & Technology (UET) Lahore Pakistan; Department of Humanities & Basic Sciences, University of Engineering & Technology Lahore, Faisalabad Campus 3½ Km. Khurrianwala – Makkuana By-Pass Faisalabad Pakistan; School of Chemical and Materials Engineering, National University of Sciences and Technology NUST H-12 Islamabad Pakistan

## Abstract

Correction for ‘Solution processed high performance perovskite solar cells based on a silver nanowire-titanium dioxide hybrid top electrode’ by Khalid Mahmood *et al.*, *RSC Adv.*, 2022, **12**, 35350–35357, https://doi.org/10.1039/D2RA06778A.

The authors regret that the name of one of the authors (Hafiz Husnain Akhtar) was shown incorrectly in the original article. The corrected author list is as shown above.

The Royal Society of Chemistry apologises for these errors and any consequent inconvenience to authors and readers.

## Supplementary Material

